# Editorial: Reviews in invasive & non-invasive ventilation in veterinary medicine

**DOI:** 10.3389/fvets.2023.1341630

**Published:** 2024-01-04

**Authors:** Liz Guieu, Céline Pouzot-Nevoret, Alexa Maria Bersenas

**Affiliations:** ^1^Department of Clinical Sciences, Colorado State University, Fort Collins, CO, United States; ^2^Université de Lyon, VetAgro Sup, Intensive Care Unit (SIAMU), APCSe, Marcy l'Etoile, France; ^3^Department of Clinical Studie, Ontario Veterinary College, University of Guelph, Guelph, ON, Canada

**Keywords:** small animal, critical care medicine, mechanical ventilation, non-invasive ventilation, respiratory failure, respiratory support

Invasive mechanical ventilation (MV) has been used for decades in veterinary medicine for the management of small animal patients with respiratory failure of various underlying etiologies. The aim of this Research Topic was to provide an overview of current knowledge and practice in the management of small animal patients requiring ventilatory support as well as to address gaps in the veterinary literature. The role of non-invasive ventilation has expanded and the introduction and recent widespread adoption of high flow nasal oxygen therapy (HFNOT) in veterinary medicine has provided a step-down ventilatory support strategy for veterinary patients. This review gathered seven articles addressing the management of small animal patients with respiratory failure. The articles provide a comprehensive analysis of the available literature on HFNOT, the application of invasive MV in various diseases processes (tick-borne disease and envenomation), optimization of the application of positive end expiratory pressure (PEEP), rescue therapies for patients with refractory hypoxemia, and nursing strategies for mechanically ventilated small animals patients.

HFNOT constitutes an augmented oxygen support modality providing heated and humidified oxygen via nasal prongs. Whitney and Keir describe the current use of HFNOT in human and veterinary medicine. In dogs with hypoxemic respiratory failure, the use of HFNOT has been well-tolerated, and for dogs failing conventional oxygen therapy, transition to HFNOT has consistently demonstrated improved oxygenation parameters in three veterinary studies (1–3). Successful weaning from HFNOT and discharge was reported in 36–66% of cases, while 27–54% died or were euthanized due to declining condition, with an additional 27% requiring escalation to MV (1–3). A case series reported the successful implementation of post-extubation HFNOT in five brachycephalic dogs to treat non-hypoxemic signs of upper airway obstruction, with the use of flow rates up to 1.5 L/kg/min for <12 h (4). Another study revealed the comparable effect of HFNOT to MV for the treatment of severe carbon monoxide intoxication in two dogs based on co-oximetry, where weaning from both the ventilator and HFNOT was successful within 4 h (5). In this section, you will also find an article from de Jaureguizar Tesas et al. reporting on the use of HFNOT during general anesthesia in four dogs undergoing bronchoscopy and bronchoalveolar lavage. The duration of bronchoscopic HFNOT ranged from 5 to 25 min and hypoxemia was limited to two events, each <1 min. The application of HFNOT in small patients requiring extubation for bronchoscopy warrants further investigation. The main reported complications in people associated with HFNOT include various degrees of hypercapnia and rare pneumothorax. Many gaps have been identified in the current veterinary literature. With higher use of HFNOT comes the need for studies investigating scoring system for early identification of patients that could benefit from HFNOT and to predict the need for escalation to MV. Optimal protocols for individual species (e.g., cats) await further exploration.

One indication for MV is hypoxemia refractory to conventional oxygen therapy. Lung protective strategies for hypoxemic patients have been extensively studied in experimental animal models and in human medicine, with results extrapolated to clinical veterinary patients. Zersen's review of PEEP optimization provides a narrative review of the many approaches to set the optimal PEEP. Current human literature recommends the use of different techniques, each with their own advantages and limitations. Incremental/decremental PEEP trials based on serial assessment of arterial oxygenation and/or evaluation of static lung compliance, setting PEEP based on pressure-volume loops, use of published PEEP tables, and evaluation of driving pressure are the most common tools used at the bedside. Rare studies evaluating the utility of these techniques were identified in veterinary medicine. Veterinary research is limited to PEEP studies of healthy canine lungs (6, 7). One study showed the benefit of PEEP in pulmonary compliance based on driving pressure (6) and another study reported no improvement in oxygenation when static compliance was used to adjust PEEP in healthy dogs (7). The author encourages clinical trials to incorporate and assess the different techniques described in more relevant small animal populations.

When conventional lung protective strategies fail to improve oxygenation, recruitment maneuvers and other rescue therapies such as prone positioning, neuromuscular blockade, and airway pressure release ventilation (APRV) should be considered, as nicely described by Bajon and Gauthier. While the physiological rational behind opening collapsed alveoli is sound, the benefits of rescue therapy are still highly controversial in ARDS human patients. Due to the heterogeneous nature of respiratory failure, an individualized approach, rather than routine use of rescue therapy, is recommended, with a need for more tools to identify appropriate candidates for each rescue therapy. Little is known in veterinary medicine regarding the utility and most appropriate way to implement these rescue therapies. Two case reports highlight the successful use of APRV for the management of hypoxemia due to aspiration pneumonia and refractory hypercapnia secondary to non-cardiogenic pulmonary edema (8, 9).

Morris and Donaldson reviewed the literature pertaining to MV in dogs and cats after snake envenomation. Neuromuscular paralysis, hypoxemia due to pulmonary hemorrhage, or aspiration pneumonia were the main indications for ventilatory support, with a median duration of mechanical ventilation of 33 h and a survival rate of 77%. O'Keeffe and Donaldson also reported the most recent evidence regarding management of dogs and cats with tick paralysis (TP). Indications for mechanical ventilation in this population include respiratory muscle failure, laryngeal paralysis, and the development of pulmonary complication (aspiration pneumonia), with a duration of ventilation ranging from 3 h to 10 days. Prognosis is overall good, with a survival rate of 53–77% in those with lung diseases and 90% in those without lung disease. Recommendations regarding ventilatory strategies for tick paralysis and snake envenomation are reflective of current best practice for MV.

While MV is invaluable for respiratory support, many complications can be associated with its application. Meitner et al. provide a comprehensive review of best nursing practices for the mechanically ventilated patient, pairing the most effective strategies reported in the human literature with the current standards in veterinary medicine. The authors identified a lack of evidence-based literature evaluating the utility of various nursing strategies in veterinary medicine.

This series offers a broad range of topics related to invasive and non-invasive ventilatory support in small animal veterinary patients that can be used as a resource at the bedside. The topics identified limited evidence-based medicine for ventilation strategies in cats and dogs, providing human recommendations where veterinary evidence is lacking.

## Author contributions

LG: Writing – original draft. CP-N: Writing – review & editing. AB: Writing – review & editing.
